# Exploring the genomic landscape of the GP63 family in *Trypanosoma cruzi*: Evolutionary dynamics and functional peculiarities

**DOI:** 10.1371/journal.pntd.0012950

**Published:** 2025-03-17

**Authors:** Luisa Berná, María Laura Chiribao, Sebastián Pita, Fernando Alvarez-Valin, Adriana Parodi-Talice

**Affiliations:** 1 Laboratorio de Interacciones Hospedero–Patógeno—UBM, Institut Pasteur Montevideo, Montevideo, Uruguay; 2 Laboratorio de Genómica Evolutiva, Facultad de Ciencias, Universidad de la República, Montevideo, Uruguay; 3 Departamento de Bioquímica, Facultad de Medicina, Universidad de la República, Montevideo, Uruguay; 4 Sección Genética Evolutiva, Instituto de Biología, Facultad de Ciencias, Universidad de la República, Montevideo, Uruguay; Baylor College of Medicine, UNITED STATES OF AMERICA

## Abstract

Members of the GP63 metalloprotease family play crucial roles in parasite-host interactions, immune evasion, and pathogenesis. Although it has been widely studied in *Leishmania* spp., less is known about its function and diversity in *Trypanosoma cruzi*. This study focuses on characterizing the complete repertoire of GP63 sequences in the *T. cruzi* genome, refining gene annotations, and exploring the evolutionary dynamics that shape the diversity of these proteins. Eleven GP63 groups were identified, which are sharply defined and have a higher intra- than inter-group sequence identity. These GP63 groups display some distinctive features. First, two groups lack an essential amino acid in the active site, indicating that they are enzymatically inactive. Second, GP63 groups show strong preference for different genomic compartments. Moreover, genes from groups located in the core genome compartment of *T. cruzi,* are often arranged as tandem arrays (of larger genomic fragments that generally include a SIRE retroposon), whereas genes from groups located in the disruptive compartment tend to be surrounded by genes encoding other surface proteins (such as MASP, mucins and trans-sialidases). Transcription patterns across different life cycle stages are not homogenous. Instead, some GP63 groups have higher mRNA levels in the infective trypomastigote stage, suggesting a potential role in host invasion. To get a wider picture of the evolutionary dynamics of these proteins, a phylogenetic analysis was conducted that included species representative of kinetoplastid diversity. It was found that 10 out of 11 GP63 *T. cruzi* groups are specific to the *Trypanosoma* genus, suggesting that the diversification of these subfamilies took place before speciation of the genus, followed by other species-specific expansions. Additionally, there are other GP63 groups that are absent in *T. cruzi*. Notably, the processes of expansion and diversification of GP63 in *Leishmania* is independent of that of trypanosomes. This suggests that these proteins may have evolved under species-specific selective (functional) pressures, resulting in unique amplifications in each parasite species.

## Author summary

In this study, the GP63 gene family was explored in the parasite *Trypanosoma cruzi*, the causative agent of Chagas disease. Eleven distinct groups of GP63 genes were identified, each occupying specific regions within the parasite’s genome. Some groups are located in the core genome, often organized into clusters, while others reside in more variable regions of the disruptive compartment, surrounded by other surface protein genes.

Our analysis revealed that the GP63 groups exhibit different patterns of gene expression, with certain groups showing higher expression during the infective stage of the parasite’s life cycle. This suggests that these proteins may play a crucial role in parasite invasion and pathogenesis. Additionally, key domains of the GP63 proteins were analyzed, revealing unique features within each group that could influence their function.

Phylogenetic studies demonstrated that most GP63 groups are exclusive to the genus *Trypanosoma*, indicating they diversified before the speciation of the genus. However, each *Trypanosoma* species has amplified certain GP63 clusters differently, following distinct evolutionary paths.

This study enhances our understanding of the diversity and evolution of these important genes, providing insights that could support the development of new strategies to combat Chagas disease.

## Introduction

Chagas disease, caused by the *Trypanosoma cruzi* parasite, affects approximately 7 million individuals worldwide and is classified as a neglected disease by the World Health Organization (WHO Chagas disease). Primarily prevalent in rural regions of Latin America, this disease holds a significant impact. The progression of Chagas disease heavily relies on the interaction of parasitic virulence factors and the defensive responses of the mammalian host. In this context, identifying parasite molecules that engage with the host’s immune or signaling systems could present novel targets crucial for disease intervention [1].

The surface of the parasite, especially during its infective stages, serves as a crucial interface for interaction with the vertebrate host. Surface proteins belonging to families such as mucins, MASPs and trans-sialidases are prominently found in this domain. While advancements have been achieved in understanding the surface architecture of this parasite [2–4], the precise roles of individual components in host-parasite interactions, as well as the dynamic expression patterns of all constituents during the infection process, remain elusive.

Following the publication of the genome sequences of the three so-called Tritryp parasites (*Trypanosoma cruzi*, *Trypanosoma brucei*, and *Leishmania major*), it became clear that each of these parasites contain gene families that encode species-specific cell-surface proteins reflecting distinct innovations associated with parasitism [5]. The Major Surface Protease (MSP) or GP63 gene family is the only surface protein component that is present across all trypanosomatid species [6]. The genes encoding GP63 proteins constitute a family that exhibits considerable variability in both number and structure across these species [7].

The GP63 protein is a metalloprotease that belongs to the group of zinc-dependent endopeptidases. Proteases related to GP63 are also found in other insect and plant parasites [7]. The diversification of trypanosomatid genomes has been accompanied by the rapid evolution of their multigene families, including the GP63 family, which underwent substantial modification after the emergence of parasitism. This is particularly evident when comparing these genomes to those of free-living kinetoplastids [6]. Phylogenetic studies of the GP63 family showed that both *T. cruzi* and *Leishmania* sp. have independently expanded their gene repertoires [6]. Therefore, this family has had a long evolutionary history, and it has been postulated that co-evolutionary processes of parasites and their hosts have significantly influenced the structure and functionality of its members [7,8].

The GP63 protein, is also known as Leishmanolysin because it was first described in *Leishmania* [9], where it has been extensively studied ever since. Although it is the most abundant protein on the surface of this parasite, some members are known to be secreted, and others remain intracellular [10]. GP63 in *Leishmania* plays several roles relevant for infection such as: inhibition of complement-mediated lysis, facilitation of phagocytosis, degradation of extracellular matrix components, alteration of host cell signaling pathways and immune evasion [10–12]. This multiplicity of functions suggests a preponderant role for parasite biology. In contrast to the extensive evidence obtained in *Leishmania*, very little is known about the functions of this family in *T. cruzi*, despite its abundance in the genome.

Alignment of several GP63 sequences from different *Leishmania* species such as *L. major*, *L. donovani*, *L.infantum* and *L.braziliensis*, shows that 60% or more of their amino acids are conserved among the different species [13]. The signature of GP63 proteins is the peptidase M8 domain (Leishmanolysin family, clan MA(M), InterPro Acc IPR001577), which comprises the majority of conserved amino acids. This domain includes the short zinc-binding motif HEXXH and 19 cysteine residues distributed along the sequence. Following synthesis, Leishmanolysin undergoes several post-translational modifications: the signal sequence is cleaved in the endoplasmic reticulum, N-glycosylation is applied, and roughly 25 C-terminal amino acids are substituted by a glycosylphosphatidylinositol (GPI) anchor. The protein is further processed by proteolytic cleavage of a propeptide, yielding the mature form. The zinc-binding motif HEXXH located in the amino-terminal domain of GP63 is the characteristic site of metalloproteases. Research demonstrates that the two histidines and one glutamic acid within this motif are conserved across all GP63 sequences in the trypanosomatids studied, playing a crucial role in proteolytic activity [14]. Mutations in any of these three residues result in a complete loss of catalytic function. Specifically, a mutation from glutamic acid to aspartic acid at position E165 in the GP63 of *L. major* leads to a loss of protease activity [15].

The variability in the GP63 gene family across *Leishmania* species, ranging from 4 to 33 genes [16], contrasts starkly with the findings in *Trypanosoma cruzi*. The hybrid strain CLBrener’s genome, initially reported in 2005 by El-Sayed et al. [5], contained 425 GP63 copies. More recently, using long-read, third-generation sequencing technologies we have provided a more accurate assembly and annotation of *T. cruzi* genomes, revealing 378 GP63 genes, including haplotypic copies, in the Dm28c strain of *T. cruzi* and 718 in the hybrid strain TCC [17]. This sequencing approach not only offered a more realistic gene count by resolving previously collapsed repetitive regions but also unveiled a compartmentalized, or ‘bipartite’, genome structure with distinct GC content and gene organization. Specifically, we previously described the *T. cruzi* genome as comprising of two different genomic compartments. One is the core compartment, which contains genes whose location and order is conserved across trypanosomatids and encodes both known and hypothetical proteins. The other is the disruptive compartment which is composed by genomic regions that are unique to *T. cruzi* and enriched with species-specific gene families such as mucins, mucin-associated surface proteins (MASPs), and trans-sialidases. Notably, GP63 family members are distributed across both genomic compartments [17]. The specific attributes of the genes within these compartments, reflecting their unique evolutionary and functional dynamics, are yet to be fully understood.

The functional role of the GP63 family of proteases in *T. cruzi* is not yet understood. Some studies showed that members of the GP63 family of *T. cruzi* may be involved in infection. This is supported by the fact that pre-incubation with specific antibodies partially blocked infection of Vero cells by trypomastigotes [18]. Other studies showed that GP63 RNAs are differentially expressed depending on the stage of the parasite life cycle [19]. A few studies on metalloproteinase activity detected on gelatin SDS-PAGE gels have been reported for a group of GP63 proteins of 75-78 kDa [18,20].

This study aims to characterize the GP63 gene family in the genome of *T. cruzi* by analyzing its subtypes, domains, genomic organization, evolutionary dynamics, expression profiles, and structural characteristics. The rationale behind this research lies in the known importance of GP63 proteins in other trypanosomatids such as *Leishmania*, including their contribution to immune modulation, tissue invasion, and parasite survival. Through this detailed analysis, we seek to contribute with knowledge necessary to elucidate the unique role of these proteins to pathogenesis and immune system evasion.

## Methods

### GP63 metalloprotease sequence dataset

We downloaded 378 sequences annotated as “surface_protease_GP63” within the *T. cruzi* Dm28c genome, from the database http://bioinformatica.fcien.edu.uy/cruzi/. Among them, 96 were identified as genes and 282 as pseudogenes.

To validate and refine these annotations, we focused on determining which sequences possessed a complete M8 peptidase domain (GP63 signature). This was performed using the InterPro predictor [21] and CDVist [22], a web server designed to maximize domain coverage in multidomain protein sequences, which enabled the identification of domains within the GP63 protein sequences. CDVist mainly uses HMMER3 search against PFAM Database to obtain specific Pfam domain hits. N-glycosilation site prediction were done using the NetNGlyc - 1.0 server that predicts N-Glycosylation sites proteins examining the sequence context of N-X-S/T sequences (https://services.healthtech.dtu.dk/services/NetNGlyc-1.0/).

The presence and location of signal peptide cleavage sites was predicted using SignalP 6.0 server [23] selecting Eukaria as an organism. The presence of a glycosylphosphatidylinositol (GPI) anchoring was predicted using two different web-servers: PredGPI [24], and NetGPI 1.1 (https://services.healthtech.dtu.dk/services/NetGPI-1.1/).

For the analysis of the conserved motif around the Cysteine of the GP63 pro-segment the motif scanning FIMO tool of the MEME suite (version 5.5.5.) was used.

### Annotation of GP63 pseudogenes

As mentioned in the previous section, the annotation of Dm28c genome includes numerous entries identified as pseudogenes. Initially, we verified if these 282 sequences did not possess a complete M8 peptidase domain, as described in the previous section. To improve the accuracy of this annotation, further evaluations were performed using BLASTN. Unlike other BLAST programs, BLASTN is unaffected by short indels, or the emergence of in-frame stop codons, which are the primary causes of pseudogenization [25]. We performed a BLASTN search against the 96 copies previously identified as functional GP63 genes (cutoff e-value: 1e-90). This search led us to discard sequences that showed no homology or had hits with less than 40% identity to the database, removing 29 sequences that had been incorrectly annotated as GP63. Among the remaining 253 sequences, which showed homology to GP63 genes, we identified 129 sequences in close genomic proximity or separated by short distances and also aligned to different regions of the same GP63 gene, suggesting that they could be parts of a single pseudogene. These sequences were further analyzed with YASS [26] which allows dotplot visualizations to clarify their structure. This approach enabled us to identify different open reading frames (ORFs) as segments of the same genes that had undergone pseudogenization. Accordingly, we re-annotated these sequences, extending them from the first nucleotide (nt) of the initial sequence to the last nt of the final constituent sequence, marking internal ORFs as derived fragments. The “new” parental pseudogene was re-annotated with an ID ending in the letter “p”.

### Cluster analysis based on amino acid identity

Identity matrix of the GP63 sequences was generated by the multiple sequence alignment tool Clustal Omega (https://www.ebi.ac.uk/Tools/msa/clustalo/) with default parameters (Gonnet matrix). Visualization of the clustering was done with Gephi (https://gephi.org/) [27], where each edge’s color tone represents the degree of identity between sequences. An edge weight threshold of 65% was applied for visualization.

Additionally, a condensed identity matrix, summarizing average intra-group and inter-group identity, was derived from mean group identity analyses. This condensed matrix was visualized using R.

### Identification of GP63 genes in other kinetoplastids and *T. cruzi* lineages

To identify GP63 genes in other kinetoplastids, we utilized publicly available genomes from TriTrypDB (release 64) [27] and NCBI. The analyzed genomes included *T. cruzi marinkellei* (B7), *Trypanosoma theileri* (Edinburgh), *Trypanosoma grayi* (ANR4), *Trypanosoma rangeli* (SC58), *Trypanosoma congolense* (IL3000), *Trypanosoma vivax* (TvY486), *Trypanosoma brucei* (TREU927), and *L. major* (Friedlin) from TriTrypDB, along with *Strigomonas culicis* (TCC012E; GCA_000482145.1), *L. braziliensis* (MHOM/BR/75/M2904; GCA_000002845.2), and *Bodo saltans* (Lake Konstanz; GCA_001460835.1), from NCBI. Annotated amino acid sequences were downloaded for these genomes.

For this analysis, we performed an iterative search using the GP63 sequences from *T. cruzi* Dm28c as the initial queries. An online BLASTP search (default parameters) was conducted against each species, selecting proteins with a query coverage >75%, which were subsequently used as queries in a second round of BLASTP with the same filter. The proteins identified were then used in a local BLASTP search against the annotated sequences of each genome, applying a filter of -evalue 1e-20, % identity >80%, query coverage >80%.

To identify GP63 genes in other *T. cruzi* lineages, we used the 96 known GP63 genes from Dm28c as queries and performed local BLASTN searches (filtering with evalue 1e-200, % of identity >80% query coverage >80) against six additional *T. cruzi* strains representing different lineages: BrazilA4, Dm25, TCC, CLBrener-Esmeraldo, CLBrenerNonEsmeraldo, and YCl6, with genomes downloaded from TriTrypDB (release 64). It should be noted that BLASTN is particularly suited to identify homologous sequences among closely related species or strains, as is the case for *T. cruzi* lineages, where DNA divergence is less than 5%, preserving the phylogenetic signal and potential drawbacks such as incomplete or lack of annotation, do not affect the identification.

### Gene environment analysis

The genomic context of each GP63 gene was analyzed by identifying the three upstream (5’) and three downstream (3’) genes to assess the gene composition surrounding GP63 loci. For GP63 genes residing in both core and disruptive compartments, we determined the most prevalent types of neighboring genes, using their annotations, processed through a custom Perl script. The results were visualized with R.

Additionally, for each GP63 gene, we estimated the distance (in bases) to the first annotated element both upstream and downstream, then calculated the average distance for GP63 genes present in the core and disruptive compartments.

### Expression profile of GP63 genes

Expression profiling for each gene was derived from the work of [28] which used RNAseq data from Dm28c. For each gene, Transcript Per Million (TPM) values from developmental stages (amastigote, epimastigote, and trypomastigote) were extracted from supplementary data [28]. Expression levels were categorized based on quartile distribution of TPM values within each stage: genes in the lowest quartile (<25%) were classified as *low*, those between the 25th and 75th percentiles were deemed *medium*, and genes in the highest quartile (>75%) were considered *high*.

### Phylogenetic analyses

The peptide sequences of the 96 GP63 genes were aligned using MAFFT [29], employing the ‘-einsi’ option for enhanced accuracy in alignments involving sequences with multiple conserved domains and gaps. Subsequent trimming was executed utilizing the ‘gappyout’ option in TrimAl [30] to remove poorly aligned regions and gaps. Phylogenetic analysis was conducted using Maximum likelihood with PhyML [31] using the JTT model with gamma distribution (exhibiting equivalent likelihood scores to other models in ModelFinder of IQ-Tree). Assessment of node reliability was inferred using 100 pseudoreplicates. Visualization of the resulting phylogenetic tree was done using iTOL [32].

To enrich the taxonomic coverage of the dataset, 282 sequences from a variety of *Trypanosoma* and *Leishmania* species, and the free living kinetoplastid *Bodo saltans*, including *T. cruzi marinkellei* (17), *T. theileri* (103), *T. grayi* (33), *T. rangeli* (55), *T. congolense* (11), *T. vivax* (12), *T. brucei* (23), *L. major* (6), *L. braziliensis* (10), *Strigomonas culicis* (3), and *Bodo saltans* (9), were incorporated. This helped provide insights into the evolutionary relationships and diversity within the GP63 gene family across these protozoan organisms.

From this expanded set of 378 sequences (282 plus 96 from *T. cruzi*), we selected representative sequences for each group of *T. cruzi* GP63 and all other clusters, totaling 103 sequences. This reduced set contains just one representative copy of each group (randomly chosen) from the large *Trypanosoma* cluster, allowing an easier visualization of the divergence outside the genus. It is worth mentioning that any other random choice of representative sequences of each group yields equivalent trees. The alignment of the selected sequences was done using MAFFT [29], retaining all positions containing information in at least 80% of the sequences. Again, phylogeny was inferred using Phyml with the Maximum likelihood method using JTT model with gamma distribution and 100 bootstrap pseudoreplicates and ITOL was used for visualization.

UTR phylogenies were performed using positions upstream and downstream of 5’ and 3’ ends, respectively. Precise determination of 5´ and 3´UTRs from GP63 genes were obtained from the UTRme tool [33], a stand-alone application to identify and annotate 5′ and 3′ UTR regions from transcriptomic data. An average length of 442 nt for the 5´ UTR and 683 nt for the 3´ UTR was determined. Consequently, 450 nt upstream and 700 nt downstream were retrieved for each of the 96 Dm28c GP63 genes. The sequences were aligned with MAFFT [29] using -auto option, and the phylogenetic tree was constructed with IQ-TREE [34] with default parameters, which in default option automatically searches for the best evolutionary model. ITOL was used for visualization.

## Results and discussion

### 1 - The metalloprotease GP63 family contains multiple functional gene copies and several pseudogenes in the genome of *Trypanosoma cruzi*

To analyze the GP63 family of metalloproteases, we used the annotated Dm28c genome sequenced with long-read technology, which is essential for including all family copies. We identified 96 complete GP63 gene sequences and 281 potential pseudogenes.

The length distribution of these sequences shows significant variation, splitting around 460 amino acids between functional sequences and pseudogenes (S1 Fig). While length can indicate functionality, it may lead to false positives. We classified a sequence as functional if it contained a full-length peptidase M8 domain and a signal peptide, since this is present in the majority of cases (see Section 4). Functional sequences exhibit marked length variability, with a bimodal distribution peaking at approximately 560 and 700 amino acids (S1 Fig).

Concerning pseudogenes, it is important to bear in mind that many sequences classified as pseudogenes may actually represent remnant fragments of the same former functional GP63 gene that underwent pseudogenization, as already reported for most trypanosomatid genes [25]. In fact, among the 281 sequences previously classified as pseudogenes, 129 are observed in pairs or groups, either juxtaposed, overlapping, or separated by short distances. Analysis of these sequences shows that they are indeed sister fragments generated by nonsense mutations or indels that cause frameshifts of a previously intact coding sequence. Segments belonging to these groups are now classified as being parts of the same pseudogene. We also found that 29 pseudogenic fragments are not really related to GP63. In all likelihood, these fragments were originally incorrectly named as GP63 because of flawed transference of information based on homology. The revised list, now comprising 185 pseudogenes, is presented in S1 Table.

It is worth noting that many of these pseudogenic fragments retain high DNA sequence identity with complete (functional) genes, suggesting their status as “young” pseudogenes, namely nonsense mutations or indels causing pseudogenization occurred relatively recently. Moreover, comparative analysis of paired pseudogenic fragments, particularly at the indels sites, shows that several pairs (or groups) contain mutations at identical positions. This pattern suggests that they are derived from duplication events that have occurred after pseudogenization (S2 Fig).

### 2 - Eleven groups of GP63 genes are present in *Trypanosoma cruzi* genome

Previously, Cuevas et al. [18] conducted PCR analyses and identified *T. cruzi* sequences homologous to GP63 genes from *Leishmania*. They categorized GP63 genes in *T. cruzi* into three groups: Tcgp63-I and Tcgp63-II, and Tcgp63-III, consisting of pseudogenes. Subsequent re-analysis of the *T. cruzi* GP63 family genes led to their classification into four distinct groups [7]. Within each group, the sequences exhibit 80% similarity, whereas between groups, the similarity drops to 30%. These authors identified a conserved segment of 100 amino acids following the active site, shared across all sequences, likely corresponding to the M8 domain, a feature ubiquitous to all GP63 sequences.

**Fig 1:**
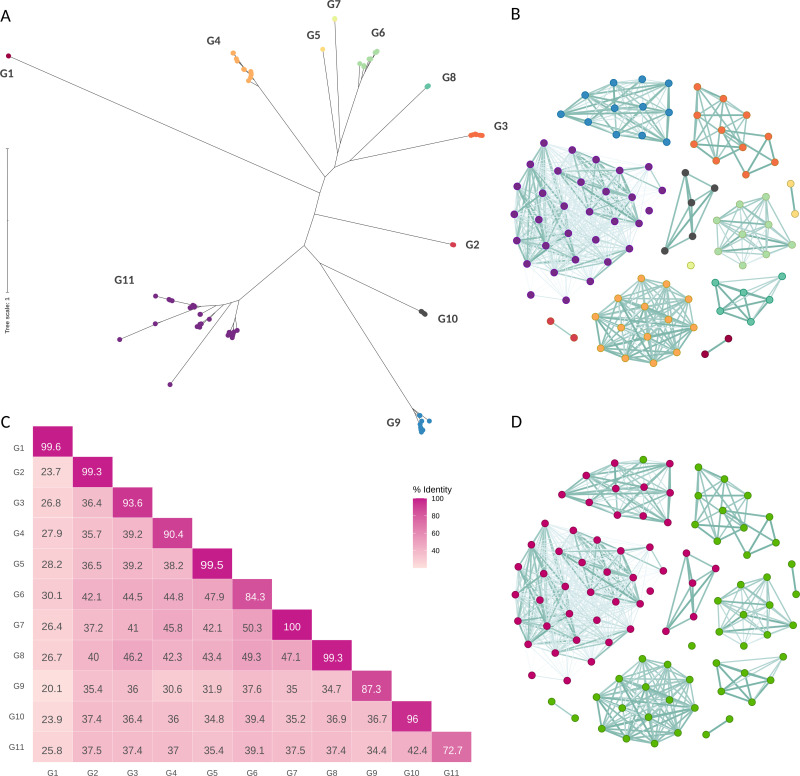
Distinct Clustering of GP63 into 11 Groups. A) Phylogenetic analysis of amino acid sequences of the 96 GP63 genes from *T. cruzi* Dm28c, illustrating the division into 11 unique groups. B) Network visualization in Gephi of the pairwise identity distances among the 96 amino acid sequences of GP63 genes, where nodes symbolize genes (color coding matches the phylogeny in A), and the color of each edge indicates the level of identity. C) Identity matrix displaying average intra-group and inter-group amino acid identities, highlighting the distinctiveness of each group. D) Network visualization similar to B, with genes colored according to the genomic compartment to which they belong: green for the core compartment and pink for the disruptive compartment.

In this study, thanks to the development of third-generation technologies that allow sequencing of increasingly better-quality long reads, new *T. cruzi* genomes, with higher accuracy have become available [17,35–37]. This advancement enabled us to conduct an exhaustive comparative analysis of the 96 annotated GP63 gene sequences, using both nucleotide and protein sequences (S2 Table). This approach led to the identification of 11 distinct groups within the GP63 gene family. Each group has a characteristic average length of sequences, which in most groups has little variation (S3 Fig). This classification is evident in a phylogenetic analysis performed with the complete amino acid sequence (Figs 1A and S4). Furthermore, an amino acid identity matrix, created through all-against-all pairwise comparisons, corroborated the observed clustering patterns (Fig 1B and S3 Table). This matrix was visualized using Gephi, where each node represents a GP63 sequence and the color of each edge denotes the level of identity, and an edge weight threshold of 65% was applied (Fig 1B).

Additionally, the identity matrix was condensed into a matrix representing average intra-group and inter-group identity, as depicted in Fig 1C. The intra-group amino acid identity levels are predominantly homogeneous, exceeding 90% in 8 out of 11 groups. In particular, three groups showed more variability, especially group G11, which is the largest and exhibited the lowest homogeneity with 73% average identity. In contrast, amino acid identities between groups were notably lower, never exceeding 48%, indicating significant divergence among groups. This pronounced divergence between groups, together with the strong homogeneity within most groups, justifies their clustering into 11 distinct groups, as highlighted in Fig 1. Furthermore, BLAST search was extended to include *T. cruzi* genes and pseudogenes. This enabled us to assign each pseudogene a parental group, thereby extending our clustering analysis beyond genes to include pseudogenes. As revealed in S1 Table, a significant proportion of these pseudogene sequences predominantly correspond to groups G9 and G11, both of which are located in the disruptive compartment. The inclusion of pseudogenes underscores the comprehensive nature of the classification, supporting the robustness of the 11-group clustering in capturing the genetic diversity within *T. cruzi*.

*T. cruzi* is a species complex composed of several lineages. A nomenclature has been proposed that organizes the multiple strains into six “discrete typing units” (DTUs) named TcI to TcVI [38,39]. More recent phylogenetic analysis using mitochondrial DNA (non-repetitive segment of maxicircles) and concatenated single copy nuclear genes from all of the DTUs, shows that *T. cruzi* is composed by four main lineages and two hybrid ones. These are clades A (TcI), B (TcIII), clade C (TcII), and clade D (TcIV), and two DTUs (TcV and TcVI) that are hybrids of strains of the BC type [40]. To determine whether the 11 GP63 groups are present in the different lineages of *T. cruzi*, we performed a BLAST search to test for their presence in genomes that are available from different strains, covering the taxonomic diversity species. Although all 11 groups are present in all lineages analyzed (S4 Table), the number of copies is not constant.

### 3 - Non-random distribution of GP63 gene groups within the *T. cruzi* genome

The genome of *T. cruzi* is composed of two clearly defined compartments, the so-called disruptive and core compartments [17]. These genomic compartments are recognized for their distinct base composition (% GC), gene content, and likely exhibit differential regulation and functions within the parasite, alongside unique evolutionary trajectories [28]. The core compartment contains genes conserved between different *T. cruzi* strains but also largely shares synteny conservation with other trypanosomatids. On the other hand, the disruptive compartment is composed almost exclusively of multigene families for surface proteins. While the majority of gene families encoding surface proteins are situated within the disruptive compartment, GP63 does not follow this pattern. The results presented here reveal that the GP63 gene family is distributed across both compartments (Figs 1D and S5). This contrasts with *Leishmania major*, which harbors only six GP63 genes in its genome with four in a single tandem array. Unlike *L. major*, *T. cruzi* shows a wide dispersion of GP63 genes throughout its genome. Notably, the genomic positioning of the GP63 groups shows a strong association with these specific genomic regions. Specifically, genes belonging to groups G1 to G8 are all found in the core compartment, whereas genes from groups G9, G10 and G11 are present almost exclusively in the disruptive compartment (Figs 1D and S5).

**Fig 2:**
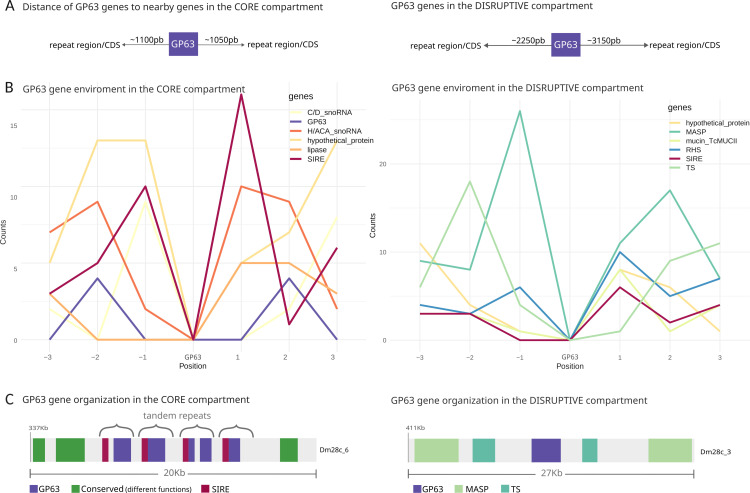
GP63 Gene Environment Composition. A) Diagram showing the average distance of GP63 genes with respect to their neighboring genes in the core (left) and disruptive (right) compartments. B) Analysis of the environment of GP63 genes within the core and the disruptive compartment illustrating the predominant genes found adjacent to each GP63 gene, focusing on the three genes upstream and downstream. C) Examples of GP63 gene environments in both compartments, with visuals sourced from http://bioinformatica.fcien.edu.uy/cruzi/browser/dm28c.500.php. For these examples, the contig and starting location of the depicted fragments are specified. Each region presents a schematic dotplot, showing the organization of the tandem repeats.

To better understand the genomic environment of the GP63 gene family within each compartment, we performed an analysis of the distance of each GP63 gene with respect to the 5’ or 3’ annotated gene or repeat sequence. In Fig 2A it can be seen that while in the core compartment such distances are on average around 1000 bp, in the disruptive compartment the average distances are greater than 2000 or 3000 bp. These results show the difference in gene density and organization between the two compartments. The lower density in the disruptive compartment may be due to the fact that this compartment contains many gene fragments from multigene families that have not been annotated.

We also performed a local gene environment analysis aiming to identify the most frequent neighbors of GP63 genes. The three most abundant genes were identified at both, 3’ or 5’ neighborhood of each GP63 coding sequence. By neighborhood we understand the three closest neighbors. We found that depending on whether the GP63 gene is located in the core or disruptive compartment, its gene environment is quite different. While genes in the core compartment mostly have a short interspersed repetitive element (SIRE)-like repeat sequence immediately upstream or downstream (Fig 2B), GP63 genes located in the disruptive compartment are found together with MASP, RHS and TS genes (Fig 2B and 2C). In addition, many GP63 genes are distributed in tandem repeats, and this is basically restricted to groups found in the core compartment (Fig 2C). In many cases, these tandem arrays do not contain exclusively the GP63 sequences but also the accompanying gene/s, meaning that the repetition unit can be somewhat larger.

The short interspersed repetitive element (SIRE)-like sequences, exclusive to *T. cruzi*, are prevalent throughout its genome, containing between 1,500 and 3,000 copies [41]. SIREs are often associated with protein-coding genes and are typically found in the 3’ untranslated regions (UTRs) of mRNAs, frequently contributing to polyadenylation sites [42]. Moreover, SIRE elements are components of VIPER, a distinctive retroelement which is presumably active in *T. cruzi*. The presence of this type of sequences in the UTRs of several of the GP63 genes may have implications in the regulation of their expression. In fact, it is known that elements present in the UTRs influence their gene expression [43]. Furthermore, the observed enrichment of SIRE elements in association with GP63 genes, especially within the core compartment, suggests their potential involvement in the gene duplication mechanisms of GP63 genes. Conversely, within the disruptive compartment, the genomic dynamics of GP63 genes are likely influenced by the amplification of other multigenic sequences, including mucins, RHS, trans-sialidases, and MASPs.

### 4 - Signal and domain recognition in predicted GP63 amino acid sequences

Peptidases of the M8 family (InterPro entry IPR001577) are defined in MEROPS database as zinc-metallopeptidases belonging to the MA clan of metallopeptidases, characterized by an active site containing a HEXXH motif involved in the coordination of the zinc ion required for catalytic activity [44]. This clan includes several subfamilies and is known for its functional diversity, including peptidases involved in extracellular protein degradation, peptide signal processing, and other critical functions in both pathogens and their hosts [44]. M8 family proteases are notably prevalent across various protozoan organisms, with *T. cruzi* boasting the largest collection of such proteins (298).

**Fig 3:**
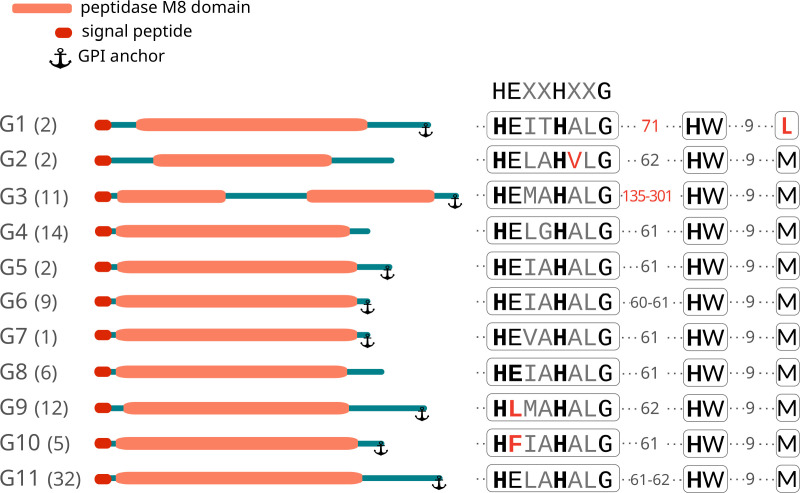
Structure of GP63 Group proteins. This figure provides a schematic overview of each GP63 group, depicting protein length, the location of the M8 domain, and the presence of signal peptides and GPI anchors. On the left side, each group is indicated and the number of sequences in each group is given in brackets. On the right side, the catalytic domain HEXXHXXG, the spacer to the subsequent histidine, and the conservation of the Met-turn are illustrated, along with all group-specific variations highlighted in red.

**Fig 4:**
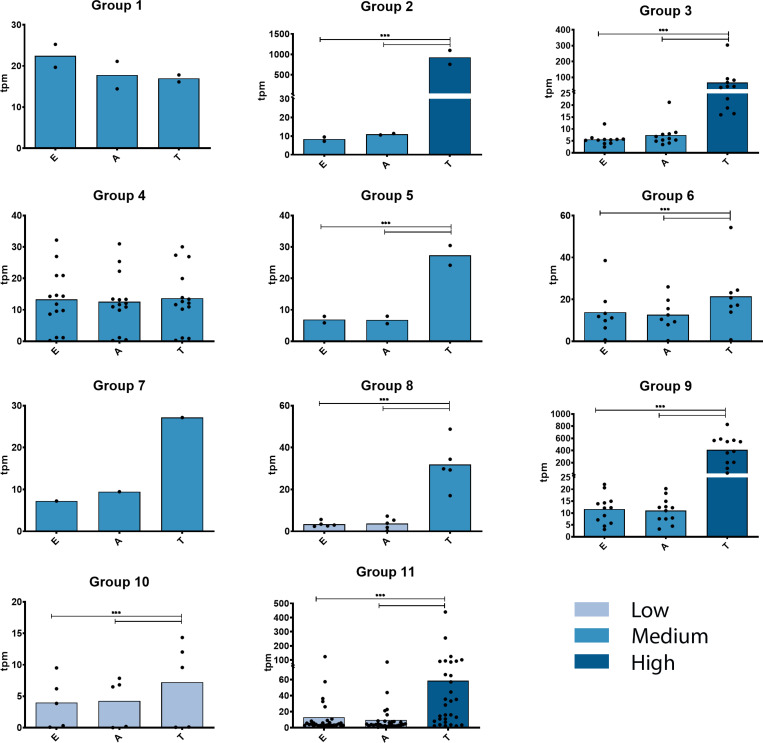
Expression profiles at the ARNm levels in the different GP63 groups. Each graph shows the expression profiles of the genes in each GP63 group. On the y-axis, expression levels are expressed in transcripts per million (tpm). Levels of expression were categorized as low, medium and high, and coloured differently. On the x-axis are the three stages of *T. cruzi*: Epimastigotes (E), Amastigotes (A) and Trypomastigotes (T). The black dots in each graph represent each individual gene. Statistical analysis of differential expression between stages was performed by one way ANOVA. *** represents a p-value <0.001.

All identified sequences were found to contain a peptidase M8 domain (Leishmanolysin family, clan MA(M), InterPro Acc IPR001577). The Metzincin clan of metalloproteinases possess a consensus sequence that includes not only the two histidines that bind the zinc ion and the general-base glutamate (HEXXH), but also an extended motif with an additional strictly conserved glycine and a third zinc-binding histidine located further along the sequence. Metzincins are also characterized by a conserved methionine-containing 1,4-β-turn, known as the Met-turn [45]. A distinctive aspect of leishmanolysins is the presence of a large insertion between the glycine and the third zinc-binding histidine. Our analysis focused on the presence of these conserved elements within the active site of the M8 domain. The zinc-binding site is preserved in most of the protein sequences, with extensive conservation observed beyond the second histidine, indicating a limited scope for variation in these positions, which consistently feature alanine and leucine (Fig 3). Intriguingly, two GP63 groups, G9 and G10, have a mutation in the highly conserved glutamic acid residue within the active site, by leucine and phenylalanine respectively. While the impact of this mutation on protein activity is unclear, it does not appear to influence gene expression. In fact, these genes exhibit RNAseq expression levels that are comparable to or exceed those of the other groups (Fig 4). Furthermore, the variable number of residues between the conserved glycine in the active site and the third histidine is striking. While most of the groups have similar or exact spacing to the *Leishmania* sequences (62 amino acids), group G3 contains a significantly larger insertion of 150 to 270 residues. These insertions split the M8 domain into two segments, significantly increasing the distance between the third histidine and the preceding two histidines. The significance of this change in protein structure is not known but would merit further study in terms of its possible functional impact. The peak around 700 aas of the distribution observed in S1 Fig is partly explained by the sequences of this group G3 with this insertion. In the case of group G1, this spacing is 71 residues. In addition, a Met-turn characterized by a highly conserved methionine is present in all groups except for this group G1. As illustrated in Fig 3, each group exhibits unique characteristics regarding these elements.

Mutations at H264, E265 and H268 of the active site of the *L. major* GP63 have been described to completely abolish catalytic function [15]. The presence of the essential active site elements in the majority of *T. cruzi* GP63 sequences suggests that these are proteins active in their protease function. In contrast, the absence of the highly conserved E residue of the active site of the sequences belonging to groups G9 and G10 of *T. cruzi*, which has been considered essential in the *L. major* protein, suggests that these two groups of sequences are not functional proteases. However, it should be noted that these proteins could have other functions, as has been described for the trans-sialidase family, a family that should be renamed because most of its members have no enzymatic activity due to a mutation in the active site [46]. However, these proteins retain other important functions by facilitating interaction with host cell receptors, promoting signaling and cell invasion of trypomastigote forms [47].

The structural analysis of GP63 amino acid sequences revealed a predicted N-terminal signal sequence across almost all GP63 variants, ranging from 23 to 37 amino acids depending on the sequence. Only in seven sequences a signal peptide could not be predicted, due in almost all cases to N-terminal deletions. Of these, four sequences corresponded to group G11, one to G9, one to G8 and one to G4. Conversely, predictions for a GPI anchor site yielded consistent results among groups. Most sequences from groups G1, G3, G5, G6, G7, G9, G10, and G11 feature a GPI anchor site, whereas sequences from groups G2, G4, and G8 appear to lack this site (Fig 3 and S1 Table). Furthermore, our examination of putative protein features, specifically the N-glycosylation sites across the 96 proteins, indicated a variability in the number of N-glycosylation sites among the groups. Notably, group G9 exhibited the highest number of glycosylation sites, as detailed in S6 Fig.

The amino- and carboxyl-terminal ends of the GP63 sequences show the greatest variability between the different members of each group, both in terms of length and sequence composition. For leishmanolysin, as well as for several other families of metzincin metalloproteinases, a “cysteine switch” mechanism has been described, based on the presence of a cysteine residue in the pro-segment that prevents access of substrates to the active site of the enzyme and thus keeps it inactive [18]. With proteolysis of the pro-segment, the enzyme would become active. A conserved motif surrounding the cysteine residue has been described [48], which in the case of GP63 from *Leishmania* sp. is HRCIHD or HRCVHD. Using the motif-based sequence analysis tools of the MEME Suite, we examined the cysteine residue at the amino-terminal ends of *T. cruzi* GP63 sequences. Our findings reveal that, except for nearly half the sequences of group G11—where the cysteine is substituted by an arginine—the cysteine residue remains conserved across all other groups where a similar motif was detectable, despite significant variations in the adjacent sequence regions (see S5 Table). An additional outlier is observed in one sequence from group G2, characterized by a deletion in this specific region.

### 5 - Expression profiles of GP63 genes

Previous studies have demonstrated the upregulated transcriptional expression of certain multigene families, such as TS [49] and MASP [3], during the trypomastigote stage (a non-replicative, infective stage interacting with the host). In contrast, the transcriptional profile of GP63 genes remains less defined. In fact, it has been suggested that while one subset of GP63 genes is minimally expressed, another exhibits increased expression in trypomastigotes, and a different subset shows elevated expression in amastigotes [50]. However, the reliability of these findings might be compromised, as these analyses were based on collapsed genomes, potentially failing to represent all gene copies accurately.

We have analyzed their expression profiles at the mRNA level with previously generated transcriptomic data from three stages of the parasite [28], observing that each group shows different expression levels and profiles and that several groups and/or members show differential expression at the trypomastigote stage compared to the epimastigote and amastigote stages. As can be seen in Fig 4, while groups G1, G4, G6 and G10 do not show differential expression profiles, the rest of the groups contain genes that are significantly expressed more in the trypomastigote stage compared to the epimastigote and amastigote stages. Remarkably, insertions in the active site of group G3 sequences do not seem to affect their expression, as they are distinctively upregulated during the trypomastigote stage, suggesting a functionality for this group. Expression levels were also very different between the groups, with those of group G2 and group G9 being the most highly expressed genes, reaching levels between one and two orders of magnitude higher than the rest of the groups (Fig 4 and S6 Table). These differential expression profiles do not correlate with the location of genes in the two genomic compartments. Groups showing differential expression are located in both the core and disruptive compartments. Moreover, the two groups with the highest expression at the RNA level are one in the core and the other in the disruptive compartment. This suggests that differential expression mechanisms are strongly linked to features associated with gene structure, e.g., possible regulatory sequence elements present in the UTRs.

### 6 - Patterns of divergence in regions embedding (surrounding) GP63 coding sequences are similar to that of proteins

**Fig 5:**
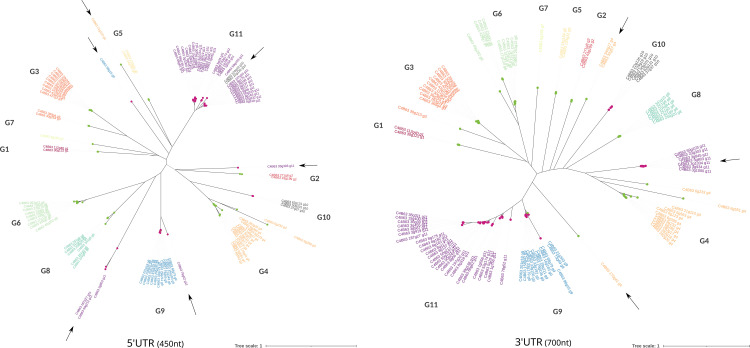
Phylogenetic Analysis of GP63 UTRs. Phylogenetic analysis of the 5’UTRs (450nt) on the left and the 3’UTRs (700nt) on the right. Groups are differentiated by color coding, and labels include the name of the respective gene and group. UTRs that do not cluster with their respective groups are highlighted with a black arrow. Additionally, nodes are colored based on the genomic compartment to which they belong.

To determine if the sequence identity observed within each GP63 group extends to untranslated regions (UTRs) or is confined solely to the coding sequence, we conducted a phylogenetic analysis paralleling the approach used for the proteins. This involved analyzing 450 nucleotides upstream (5’ UTR) and 700 nucleotides downstream (3’ UTR) of each GP63 gene, which represents the average length of the UTRs present in the GP63 genes. As illustrated in Fig 5, 5’ and 3’ UTRs largery mirror those seen in the phylogenetic analysis of the proteins.

5’ and 3’ UTRs largely mirror those seen in the phylogenetic analysis of the proteins. This suggests that GP63 genes within each group not only share sequence identity in their coding regions but also in their UTRs, with the similarity being more pronounced in the 3’ UTR than in the 5’ UTR. As previously discussed, tandem duplication represents a form of gene amplification within this family, with conservation in these regions indicating that duplication encompasses more than just the coding sequence. Moreover, the UTRs, particularly the 3’UTR, are pivotal in gene expression regulation [51]. Hence, the conserved clustering of these sequences might suggest a differential expression regulation, potentially varying across developmental stages or in response to different stress conditions. Notably, in the 3’UTR phylogeny, group G4 undergoes a division, with three sequences diverging from this group (indicated by an arrow in Fig 5). These sequences are the 3’UTRs of genes C4B63_170g41, C4B63_320g17, and C4B63_32g57, which represent the non-expressed genes of G4 at any developmental stage (see Fig 4 and S6 Table for reference). Similarly, group G11 bifurcates, with 7 out of the 32 3’UTRs clustering separately. The genes corresponding to these 3’UTRs show very low expression levels during the trypomastigote stage, in contrast to the remaining genes, which exhibit increased expression during this stage.

Taken together, these results suggest that this family of proteins with different levels of mRNA expression may be strongly regulated at the post-transcriptional level by elements present in the UTRs, as has been described for *Leishmania* genes [48].

### 7 - Amplification of GP63 genes is partially mirrored in other trypanosomes

**Fig 6:**
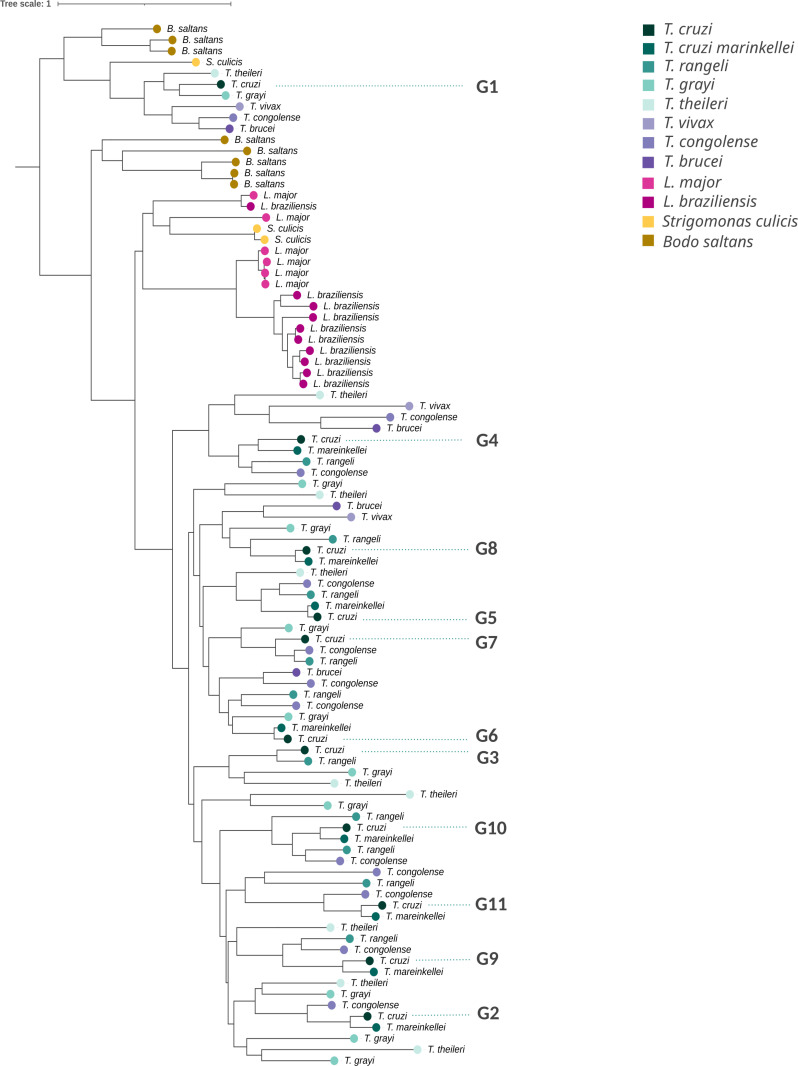
Phylogenetic Analysis of representative GP63 Genes Across Kinetoplastids. Phylogenetic analysis using PhyML (Maximum Likelihood method) based on the JTT model with gamma distribution and 100 bootstrap pseudoreplicates. It focuses on the entire GP63 amino acid sequences from representative genes of *T. cruzi, T. cruzi marinkellei, T. rangeli, T. grayi, T. theileri, T. vivax, T. congolense, T. brucei, Leishmania major, L. braziliensis, Strigomonas culicis*, and *Bodo saltans*.

**Fig 7:**
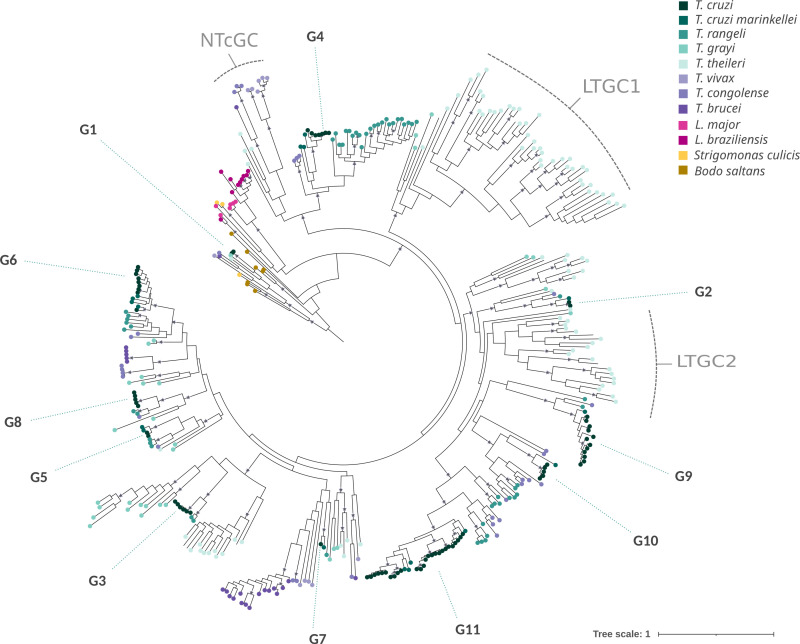
Global Phylogenetic Analysis of GP63 Genes Across Kinetoplastids. Phylogenetic analysis of an enriched dataset of 282 sequences from a diverse array of kinetoplastids, i.e.,: *T. cruzi, T. cruzi marinkellei, T. rangeli, T. grayi, T. theileri, T. vivax, T. congolense, T. brucei, L. major, L. braziliensis, Strigomonas culicis*, and *Bodo saltans*. Pseudoreplicates support values above 75 are indicated at the respective nodes.

The eleven groups described in section 2, most likely reflect functional diversification in the GP63 family in *T. cruzi*. It is of great interest to determine whether this divergence is observed in other species of the genus *Trypanosoma*, other trypanosomatids and even kinetoplastids outside the trypanosomatidae family. For this purpose, we conducted a phylogenetic analysis including all completed GP63 sequences identified in eight *Trypanosoma* species, covering the taxonomic diversity of the genus *(Trypanosoma cruzi*, *T. cruzi marinkellei*, *Trypanosoma rangeli*, *Trypanosoma theileri*, *Trypanosoma grayi*, and three african trypanosomes). We also included the genus *Strigomonas* (*Blastocrithidia*), two *Leishmania* species (*Leishmania major* and *Leishmania braziliensis*) and the free living kinetoplastid *Bodo saltans.* This taxonomic sampling is a good representation of the diversity of kinetoplastids [6]. The two phylogenetic trees presented in Fig 6 and Fig 7 allow us to highlight different aspects of this divergence. These two trees differ in that Fig 6 contains just one representative copy of each group (randomly chosen) from the large *Trypanosoma* cluster, allowing an easier visualization of the divergence outside the genus. From Fig 6 it is evident that ten of the eleven groups described above represent *Trypanosoma* specific subfamilies. Namely, genes belonging to these groups are exclusively found in species of this genus, whereas homologues to group G1 are observed outside the genus. Interestingly, members of the G1-like subfamily are found as single copy genes not only in *T. cruzi* but in all species of *Trypanosoma* and *Strigomonas*. The gene is also present in *B. saltans* as multicopy but absent in *Leishmania*. The phylogenetic location of G1-like group, as the earliest branching lineage, and its widespread presence in kinetoplastids is indicative of its ancestrality. This, in turn, is reflected in two aspects of its biology that are worth mentioning. First, it is the only group that lacks the conserved Methionine of the Met-turn motif (see Fig 3) having instead a Leucine. The presence of this non-canonical Leucine at this site is not only observed in *T. cruzi*, but also in G1-like genes from other kinetoplastid species (i.e., orthologs to *T. cruzi* G1). Second, in *T. cruzi,* the G1 group is transcribed at constant levels throughout the life cycle, suggesting an absent or weak relationship with the infective stage, a feature that is compatible with its presence in the non-parasitic kinetoplastid *B. saltans*.

Another interesting aspect from Fig 6, is the pattern of amplification and divergence of GP63 in both *L. major* and *L. braziliensis*. The interest in understanding the pattern of divergence in these two species and its comparison with *T. cruzi* is fundamentally due to the fact that this is a classic and extensively studied *Leishmania* protein (in fact it is called leishmanolysin). Noteworthy, there are only three relatively divergent gene clusters, one of which has 9 copies in *L. braziliensis* and 4 in *L. major* [52]. The other two clusters were not expanded. Two relevant observations in relation to these *Leishmania* clusters can be drawn from Figs 6 and 7. Indeed, copies from these clusters are not represented in any species of the genus *Trypanosoma* (nor in *Bodo saltans*). Moreover, the degree of amino acid divergence among copies in the expanded *Leishmania* cluster is very modest, this being true for both *Leishmania* species included in this study (*L. major* and *L. braziliensis*). Besides, similarities are higher within, than among species. The latter may indicate that the amplifications emerged independently (i.e., are species specific), or that there is a high rate of gene conversion. We note that in spite of this comparatively low copy number and differentiation within this cluster in *Leishmania,* GP63 is able to exert different functions, facilitating adhesion and invasion and cleaving or degrading several host cell proteins that play important roles in the cellular response, such as transcription factors and components of signaling cascades [11].

Fig 7 is basically an enlargement of Fig 6 that incorporates the intragroup variability within the eight species of the genus *Trypanosoma* (378). As previously noted, ten of the eleven *T. cruzi* groups, defined in section 2, form part of this large clade. A relevant observation is that members of these ten groups are also found in other *Trypanosoma* species, including African ones. There are some exceptions though: group G3 is absent in African trypanosomes, and groups G4 and G9 are not found in *T. grayi* and *T. theileri*. The copy number of each group exhibits considerable variation across different species, with group G11 notably expanded in *T. cruzi*. In general, *T. cruzi* exhibits very high intra-cluster similarity, in contrast to what is observed in other species of *Trypanosoma* genus. In this regard, the pattern of *T. cruzi* is, to some extent, similar to that of *Leishmania.* Besides the groups already described in *T. cruzi*, this large trypanosome-exclusive clade contains additional large clusters that are present in other species but have no representation in *T. cruzi*. One of such clusters, designated LTGC1 (Large Theileri Gp63 Cluster 1), includes over 35 copies in *T. theileri*, with a few distant relatives in *T. grayi*. A second notable expansion is observed in *T. theileri*, termed LTGC2 (Large Theileri Gp63 Cluster 2), which comprises approximately 20 copies. Genes within these non-*T.cruzi* GP63 groups also possess a complete M8 domain and signal peptide, suggesting their likely functionality.

The two large *Theileri* cluster (LTGC1 and LTGC2), most probably represent species specific expansions, rather than two independent losses in *T. cruzi* (and its nearest neighbors, *T.c marinkellei* and *T. rangeli* [6]) and Salivaria (african trypanosomes). These clusters contain long branches, indicative of faster evolutionary pace, probably reflecting specific functional requirements. Many gene losses are also apparent. A few examples are the clade containing *T.cruzi*-G9 and relatives (G9-like), which is absent in *T. brucei* and *T. congolense*, but present in *T. vivax* (indicating it was present in the Salivaria ancestor) as well as *T. theileri*. Likewise, G6- and G5-like, are absent in *T. brucei* and *T. congolense*, but present in *T. vivax*. The last loss we would like to mention is the absence of *T. cruzi* and closest relatives (*T.c marinkellei* and *T. rangeli)* from a group of sequences labeled as N*Tc*GC (Non *T. cruzi* GP63 Cluster) in Fig 7, which is found in the remaining species of the genus *Trypanosoma.*

## Conclusions

In this study we address the amplification and diversification of GP63 genes in the genome of *T. cruzi*, using as an initial reference our previous genomic studies on the Dm28c strain. The analysis identified the existence of eleven groups, each distinguished by specific characteristics such as sequence length, unique features within the conserved elements of the M8 motif and different expression profiles. While there is remarkable homogeneity within each group, substantial divergence is observed when comparing sequences across different groups. Notably, these groups exhibit variation in the presence of GPI anchor signals in the C-terminal regions, and in the number of sites for N-glycosylation.

Furthermore, we found a strong correlation between these GP63 groups and the core and disruptive genomic compartments of the *T. cruzi* genome. Multi-copy tandem arrays of GP63 genes are prevalent in groups situated in the core compartment, whereas those in the disruptive compartment are typically flanked by genes from other multigene families, such as mucins, MASPs, and trans-sialidases.

This study also provides insights into the evolutionary history of the GP63 gene family, in particular the expansions, retractions and losses of the family in *Trypanosoma, Leishmania* and relatives. Phylogenetic analysis indicates that while only the group G1-like is present in most trypanosomatidae species and the nonparasitic kinetoplastid *Bodo saltans* (indicating that the group G1 represents the earliest branching lineage), the GP63 gene family has undergone some (probably independent) large expansions. One of these expansions occurred in *Leishmania*, but the most prominent one is that of the genus *Trypanosoma* where 10 of the 11 groups characterized in this study are shared only within the genus *Trypanosoma* and are present in most species, including African trypanosomes. Besides, these 10 groups (and others like LTGC1 and LTGC2) not only are *Trypanosoma*-exclusive but also they constitute a monophyletic clade. Their widespread presence in the genus and its monophyletic status reveals that its expansion occurred prior to the separation of *Trypanosoma* species.

While this study provides valuable insights into the diversity, organization, and expression of GP63 genes in *T. cruzi*, several inferences presented here from in silico-analyses should require experimental validations. Furthermore, the reliance on mRNA expression data alone to infer functional roles has its limitations, as expression levels may not directly translate to protein abundance or activity *in vivo*.

Experimental validation, such as functional assays, protein localization studies, or host-parasite interaction models, will be essential to confirm the predicted roles of GP63 proteins in immune evasion and pathogenesis. Future studies integrating biochemical and cellular experiments will be necessary to build a more comprehensive understanding of these proteins and their potential as therapeutic targets.

## Supporting information

S1 Fig Distribution of GP63 amino acid sequence lengths in *T. cruzi* Dm28c. This figure displays two distinct length distributions: on the left, the length distribution of GP63 functional genes and on the right, the length distribution of GP63 pseudogenes.
(PDF)

S2 Fig Reassignment of pseudogene fragments, presenting two examples at the top. In these instances, alignments were conducted using the YASS tool, with the C4B63_1g558 gene serving as the reference. Below, the figure provides a visualization of the pairwise local alignments for four pseudogene pairs, demonstrating the consistent pattern observed in the examples above.
(PDF)

S3 Fig Average length of the amino acid sequences for each GP63 group, including the standard deviation. (PDF)

S4 Fig Phylogenetic analysis of the 96 GP63 amino acid sequences from *T. cruzi* Dm28c. Groups are differentiated by color coding in labels. Nodes are colored based on the genomic compartment, core in green, disruptive in purple.
(PDF)

S5 Fig Genomic distribution of GP63 gene sequences across *T. cruzi* groups. Green color corresponds to groups present in the core compartment, purple to groups in the disruptive compartment.
(PDF)

S6 Fig Average number of predicted N-glycosylation sites in each group of GP63 sequences. (PDF)

S1 Table List, length and composition of annotated GP63 pseudogenes. (XLSX)

S2 Table List of GP63 genes, including descriptive statistics. (XLSX)

S3 Table Amino acid identity matrix among all GP63 genes. (XLSX)

S4 Table GP63 in *T. cruzi* lineages. (XLSX)

S5 Table Cys switch motif search. (XLSX)

S6 Table Gp63 expression in different *T. cruzi* stages. (XLSX)

S7 Table List of FASTA GP63 genes. (XLSX)
