# Supplementary figures and images for: Exploring the genomic landscape of the GP63 family in Trypanosoma cruzi: Evolutionary dynamics and functional peculiarities
Source: PLoS Negl Trop Dis. 2025 Mar 17;19(3):e0012950. doi: 10.1371/journal.pntd.0012950 (PMC11957388; doi:10.1371/journal.pntd.0012950)

GP63 genes and pseudogenes

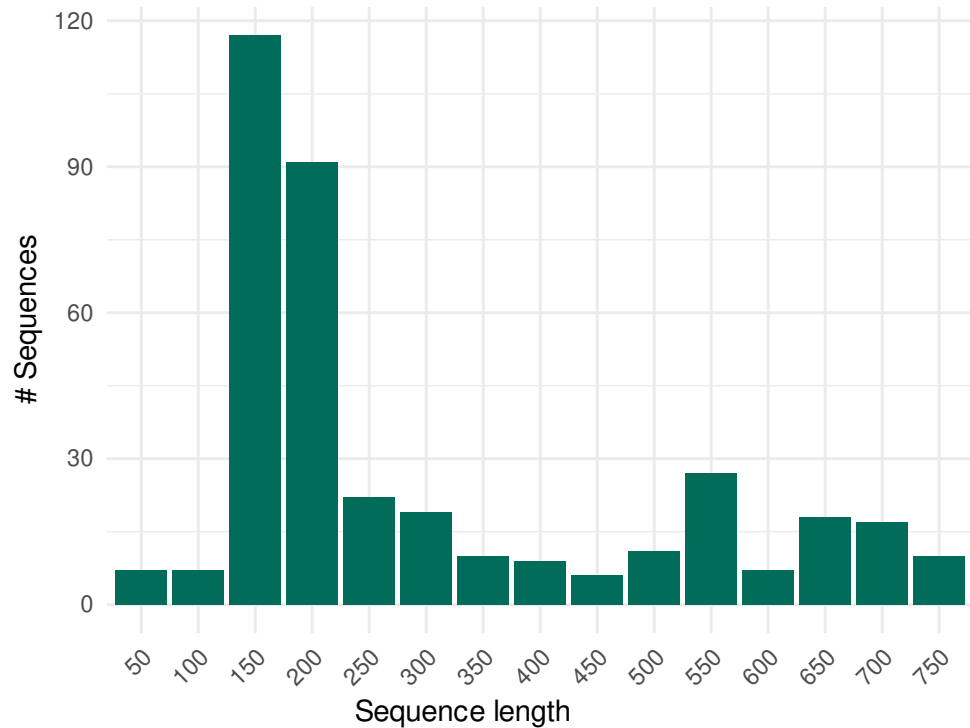

GP63 genes

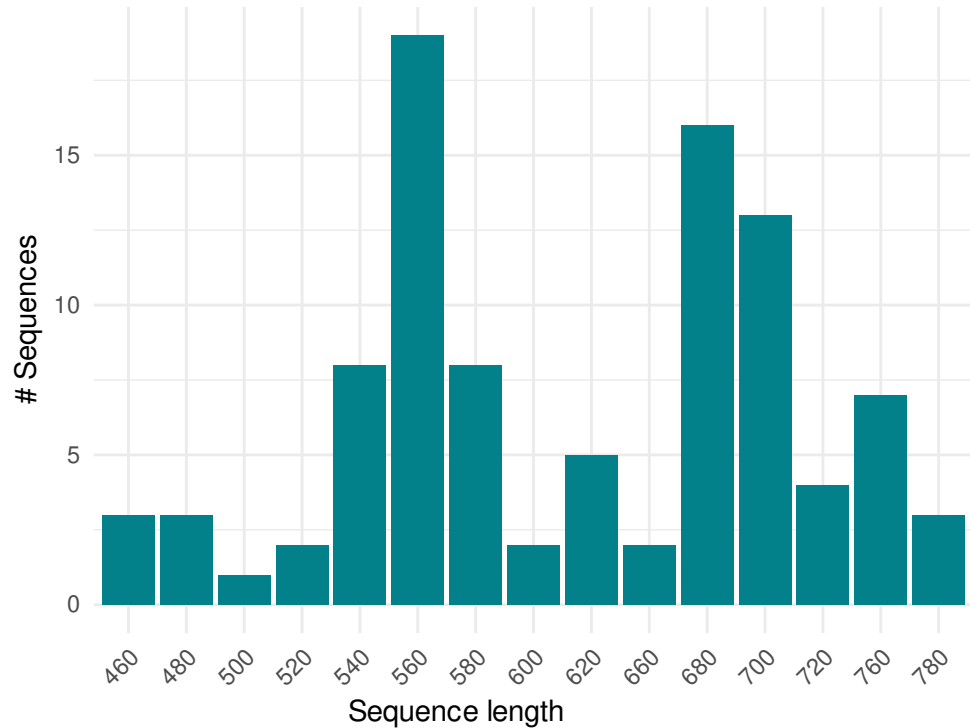

Supplement: S1 Fig — This figure displays two distinct length distributions: on the left, the length distribution of GP63 functional genes and on the right, the length distribution of GP63 pseudogenes. (PDF) [file pntd.0012950.s001.pdf]

C4B63\_1g558

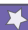

C4B63\_1g227

C4B63\_1g228

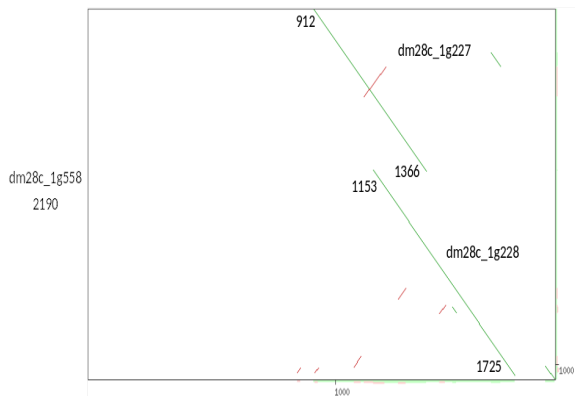

C4B63\_1g558

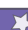

C4B63\_1g403

C4B63\_1g404

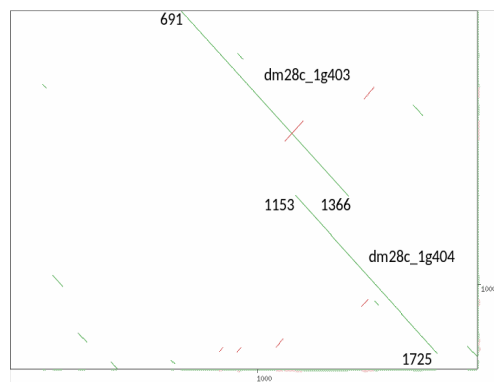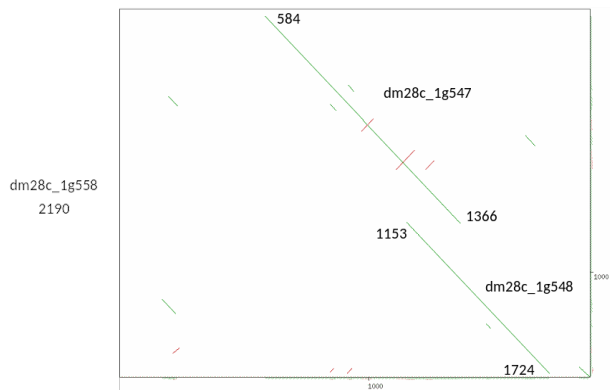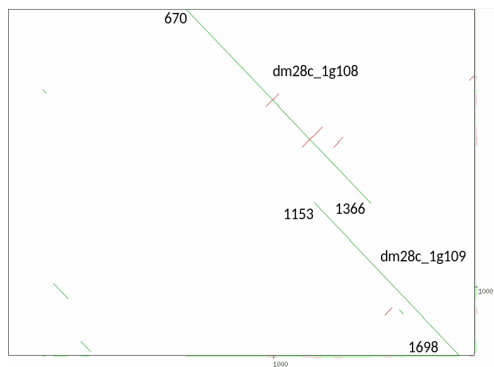

Supplement: S2 Fig — In these instances, alignments were conducted using the YASS tool, with the C4B63_1g558 gene serving as the reference. Below, the figure provides a visualization of the pairwise local alignments for four pseudogene pairs, demonstrating the consistent pattern observed in the examples above. (PDF) [file pntd.0012950.s002.pdf]

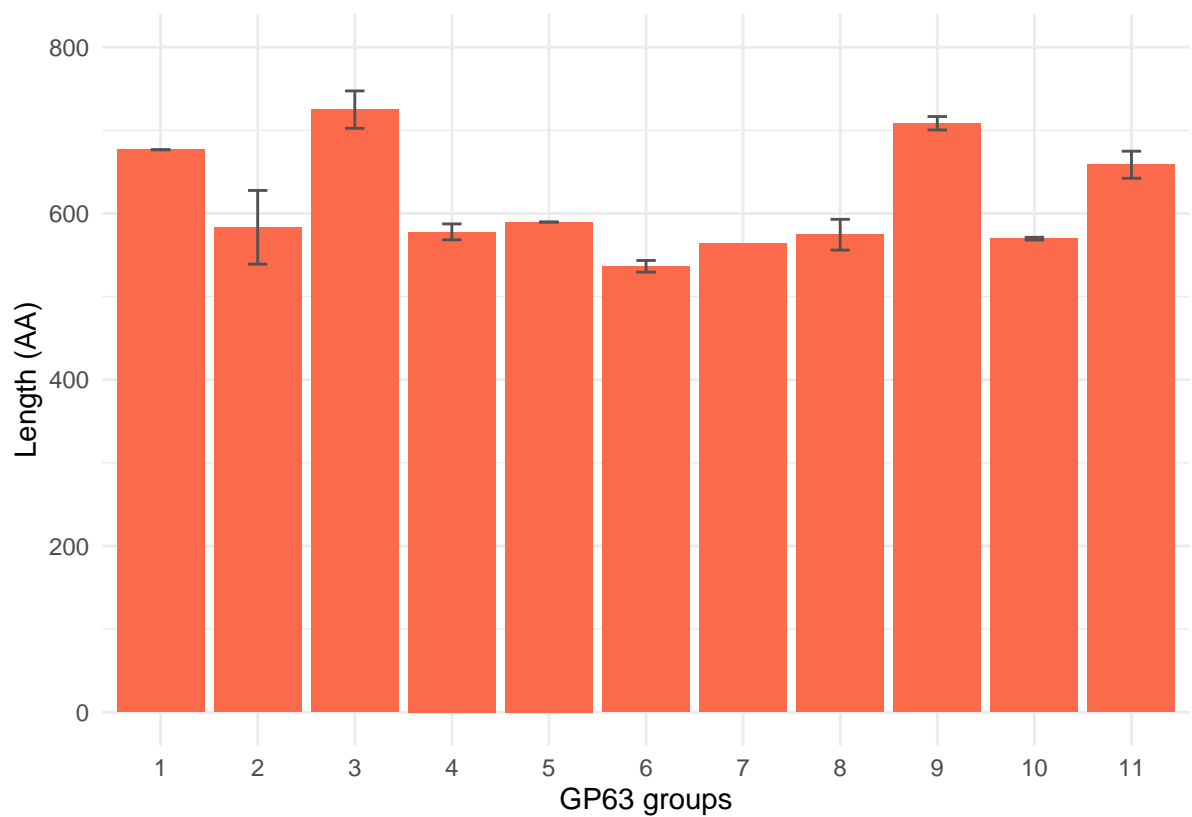

Supplement: S3 Fig — (PDF) [file pntd.0012950.s003.pdf]

GP63 in *T. cruzi* Dm28c by group

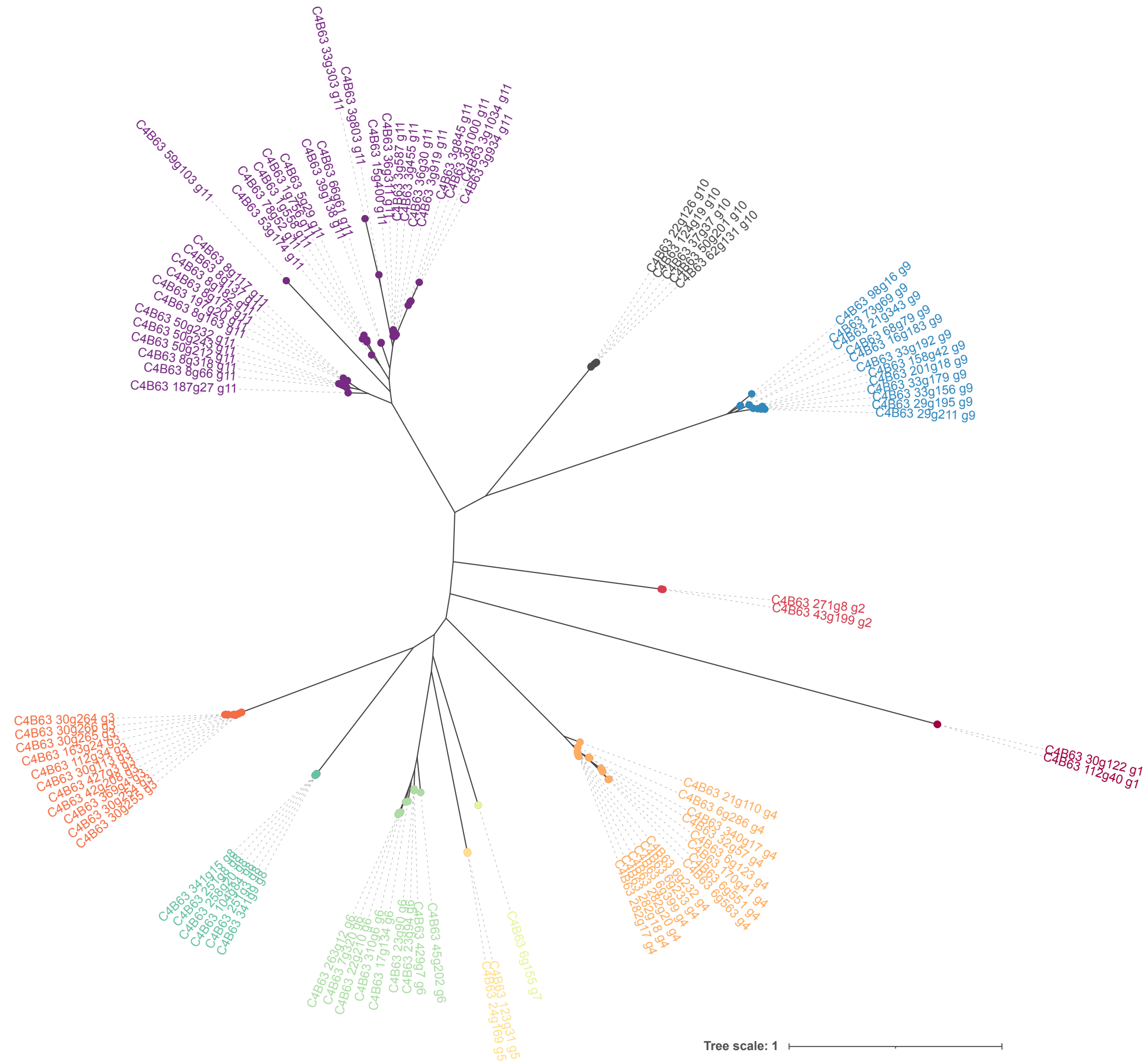

### GP63 in *T. cruzi* Dm28c by compartment

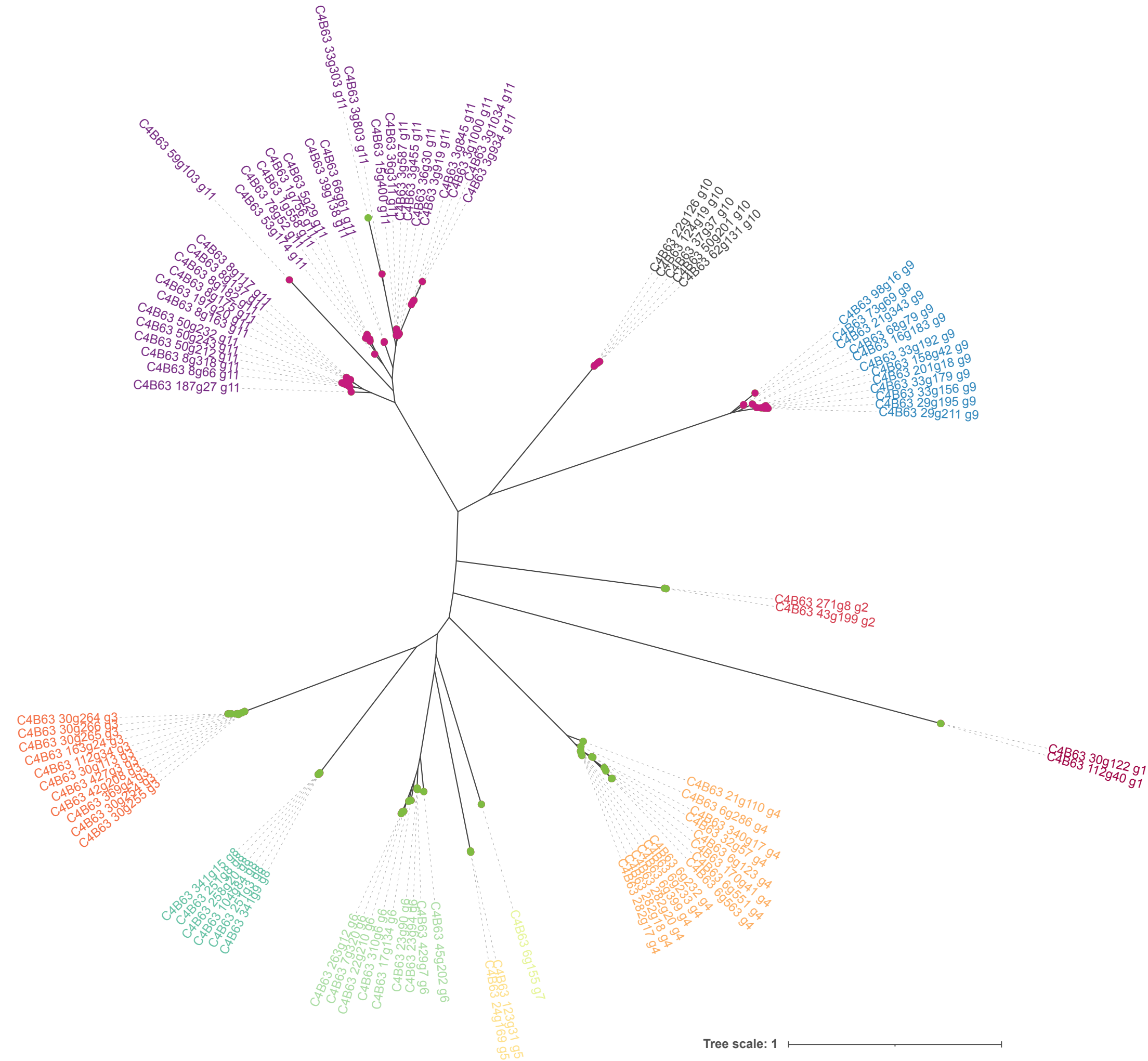

Supplement: S4 Fig — Groups are differentiated by color coding in labels. Nodes are colored based on the genomic compartment, core in green, disruptive in purple. (PDF) [file pntd.0012950.s004.pdf]

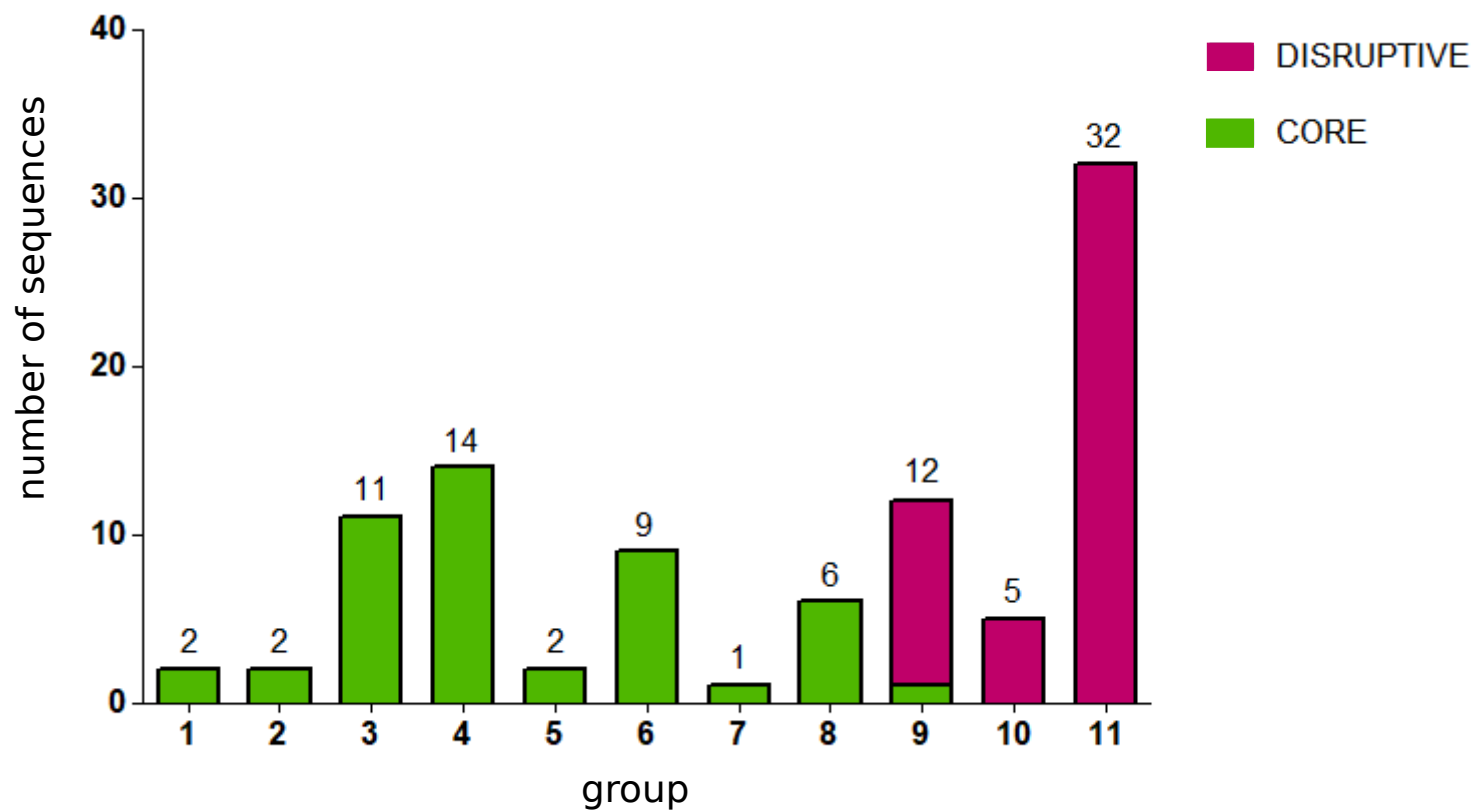

Supplement: S5 Fig — Green color corresponds to groups present in the core compartment, purple to groups in the disruptive compartment. (PDF) [file pntd.0012950.s005.pdf]

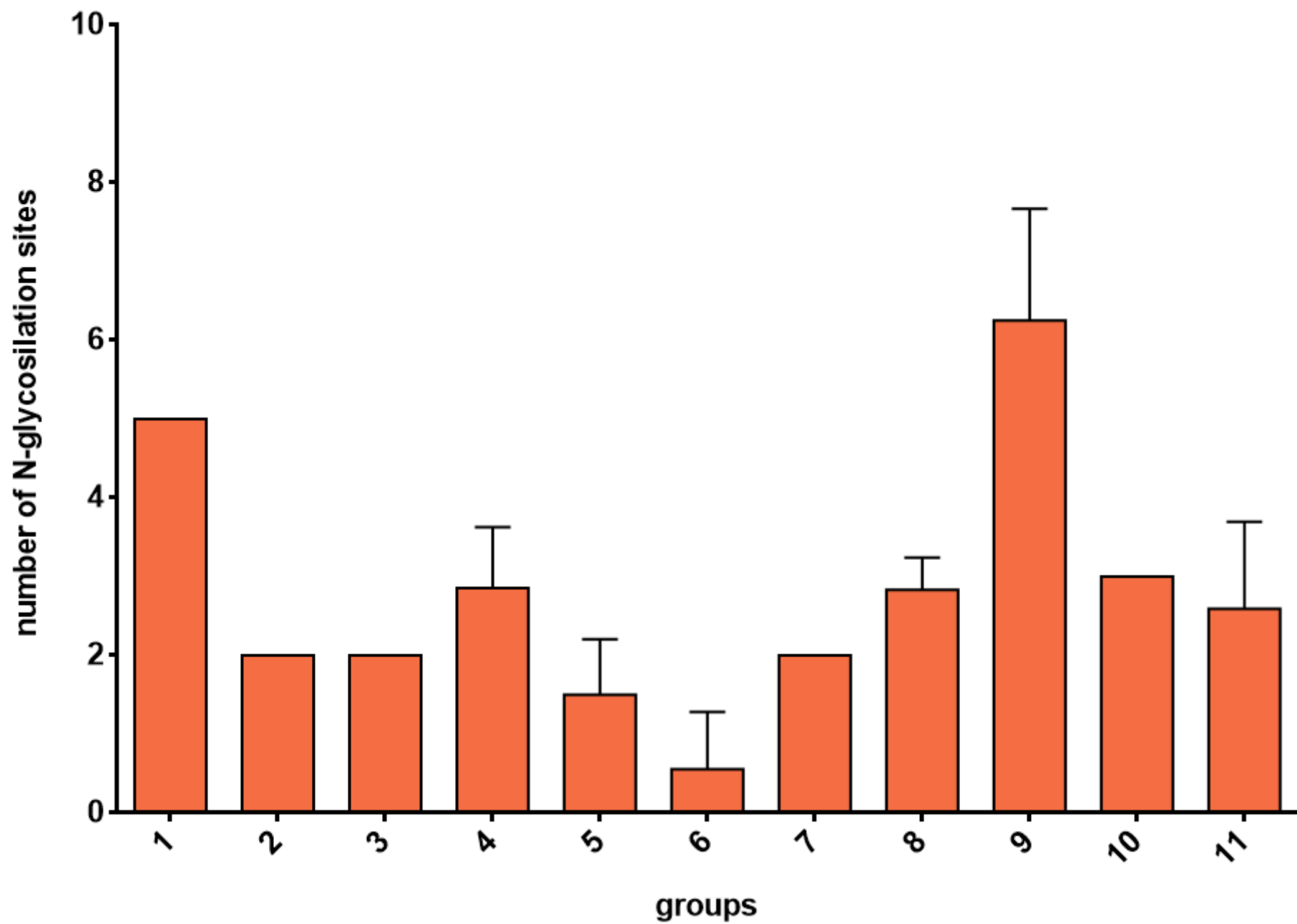

Supplement: S6 Fig — (PDF) [file pntd.0012950.s006.pdf]
